# QTL for white spot syndrome virus resistance and the sex-determining locus in the Indian black tiger shrimp (*Penaeus monodon*)

**DOI:** 10.1186/1471-2164-15-731

**Published:** 2014-08-28

**Authors:** Nicholas A Robinson, Gopalapillay Gopikrishna, Matthew Baranski, Vinaya Kumar Katneni, Mudagandur S Shekhar, Jayakani Shanmugakarthik, Sarangapani Jothivel, Chavali Gopal, Pitchaiyappan Ravichandran, Thomas Gitterle, Alphis G Ponniah

**Affiliations:** Nofima, PO Box 210, 1431 Ås, Norway; Central Institute of Brackishwater Aquaculture, Raja Annamalai Puram, Chennai, 600028 Tamil Nadu India; Akvaforsk Genetics Centre, Sjølseng N-6600 Sunndalsøra, Norway; Flinders University, Sturt Road, Bedford Park, SA 5042 Australia

**Keywords:** *Penaeus monodon*, Single nucleotide polymorphism, White spot syndrome virus, Disease resistance, Sex-linked gene markers, Quantitative trait loci

## Abstract

**Background:**

Shrimp culture is a fast growing aquaculture sector, but in recent years there has been a shift away from tiger shrimp *Penaeus monodon* to other species. This is largely due to the susceptibility of *P. monodon* to white spot syndrome virus disease (*Whispovirus sp*.) which has impacted production around the world. As female penaeid shrimp grow more rapidly than males, mono-sex production would be advantageous, however little is known about genes controlling or markers associated with sex determination in shrimp. In this study, a mapped set of 3959 transcribed single nucleotide polymorphisms were used to scan the *P. monodon* genome for loci associated with resistance to white-spot syndrome virus and sex in seven full-sibling tiger shrimp families challenged with white spot syndrome virus.

**Results:**

Linkage groups 2, 3, 5, 6, 17, 18, 19, 22, 27 and 43 were found to contain quantitative trait loci significantly associated with *hours of survival* after white spot syndrome virus infection (*P* < 0.05 after Bonferroni correction). Nine QTL were significantly associated with *hours of survival*. Of the SNPs mapping to these and other regions with suggestive associations, many were found to occur in transcripts showing homology to genes with putative immune functions of interest, including genes affecting the action of the ubiquitin-proteasome pathway, lymphocyte-cell function, heat shock proteins, the TOLL pathway, protein kinase signal transduction pathways, mRNA binding proteins, lectins and genes affecting the development and differentiation of the immune system (eg. RUNT protein 1A). Several SNPs significantly associated with sex were mapped to linkage group 30, the strongest associations (*P* < 0.001 after Bonferroni correction) for 3 SNPs located in a 0.8 cM stretch between positions 43.5 and 44.3 cM where the feminisation gene (FEM-1, affecting sexual differentiation in *Caenorhabditis elegans*) mapped.

**Conclusions:**

The markers for disease resistance and sexual differentiation identified by this study could be useful for marker assisted selection to improve resistance to WSSV and for identifying homogametic female individuals for mono-sex (all female) production. The genes with putative functions affecting immunity and sexual differentiation that were found to closely map to these loci provide leads about the mechanisms affecting these important economic traits in shrimp.

**Electronic supplementary material:**

The online version of this article (doi:10.1186/1471-2164-15-731) contains supplementary material, which is available to authorized users.

## Background

Crustaceans make up around 10% of the world’s aquaculture production with average growth in production of 15% per year from 1970 reaching 5 million tonnes in 2008 [1]. Rapid growth during the period 2001–2008 was due to increased production of *Litopenaeus vannamei* in China, Thailand, and Indonesia. Production of *P. chinensis* has been reduced, and no significant change in the production of *P. monodon* has occurred over the last 13 years, mainly because of difficulties due to disease with white spot syndrome virus in these species and the increased availability of genetically improved specific pathogen free *L. vannamei* post-larvae. More than 80% of shrimp exports from India are derived from aquaculture production.

One of the major worldwide problems limiting the culture of shrimp is viral disease. White spot syndrome virus (family Nimaviridae, genus *Whispovirus*, WSSV) is a lethal pathogen that can cause up to 100% mortality within 7–10 days on shrimp farms, and has devastated shrimp farming industries across the world (reviews by: [2–4]). Selective breeding has been suggested by many as a highly effective long term strategy to combat the threat of disease [5]. However, resistance to WSSV has low heritability in *L. vannamei* [6–11], and limited evidence has been found for genetic variation in resistance to WSSV in *P. monodon* [12, 13], especially because of the difficulty with developing a standardized challenge protocol for WSSV. Shrimp exposed to WSSV have a rapid mortality rate and cannibalism can cause secondary waves of infection. Oral infection of individual shrimp with a controlled dose of the virus, although technically difficult and labour intensive, is recommended [8]. Where genetic resistance has been detected, it has been found to be strongly negatively correlated with growth rate [10].

Shrimp have a very limited adaptive immune response [14] and lack diverse immune related molecules such as immunoglobulin, T cell receptor and major histocompatibility complex. The innate immune response of shrimp has been shown to be triggered almost instantaneously in response to peptidoglycan stimulation [15] and is believed to be the primary defence mechanism against infection in this group of species. A number of potential antimicrobial peptide coding genes have been isolated from penaeid shrimp and some such as penaeidins and crustins have been found to be differentially expressed over the time course of infection [16–18]. The susceptibility of *P. monodon* to white spot disease has been shown to increase when penaeidin class 5 expression is suppressed by interference mediated gene silencing [19]. Shrimp surviving 84 hours post-infection have higher expression of lysozyme, C-type lectin, penaeidins, prophenoloxidase-1 and prophenoloxidase-2 in haemocytes than those dying less than 60 hours post infection [18]. Heat shock protein 21 is down regulated after infection to WSSV [20]. Shrimp lysozyme has been shown to be effective in blocking infection by WSSV in blue shrimp (*Litopenaeus stylirostris*) [21].

As yet there are no vaccines or other treatments available with proven efficacy against WSSV, although a number of studies have revealed promising leads. The WSSV binding proteins isolated from viral particles in the haemolymph of shrimp infected with WSSV, have been shown to inhibit the binding of this virus to haemolymph cells and improve survival of shrimp [22]. Injection of shrimp with recombinant fortilin after infection with WSSV, results in 80-100% survival and low levels of WSSV are detected, suggesting that fortilin inhibits viral replication [23]. Fortilin is highly upregulated in haemolymph during the early phase of white spot infection [24]. Injection with recombinant ferritin or lysozyme also results in protection to challenge with WSSV [21, 25]. Inoculation in feed with bacterially expressed double stranded RNA VP28 (encoding for an envelope protein found in WSSV) and vaccination with VP28 and recombinant VP292 [26–29], as well as exposure to probiotics and beta-1,3/1,6-glucans [30], have been shown to provide improved survivability. Shrimp immunity to WSSV was shown to be enhanced by intramuscular injection of oligodeoxynucleotides with Cytosine-Guanine motifs and *Vibrio harveyi* DNA [31]. In addition, double stranded RNA of any type has been found to induce antiviral protection in shrimp [32]. Interestingly, a gene designated as PmAV was isolated using differential display from viral resistant shrimp and was shown to have antiviral activity [33].

Resistance to WSSV is a strong candidate trait for marker-assisted or genomic selection since it appears to have low heritability and has a negative correlation with another selected trait (growth). The lack of reported quantitative trait loci associated with this trait may not be due to the lack of segregating genes for resistance, but could instead be due to the highly virulent nature of WSSV, challenge testing methods that do not deliver accurate resistant phenotypes and because marker resources do not sufficiently cover the genome.

Another important factor in shrimp cultivation is sex determination. Female penaeid shrimps grow more rapidly than males and so mono-sex production of females would be advantageous for production [34]. This could also be used to provide a level of genetic protection, hindering the replication of genetically superior stock. In penaeid shrimps, females are known to be heterogametic with sex determined by a WZ-ZZ chromosomal system [35–37]. However, more detailed mapping studies are needed to find closely linked markers and genes associated with sex determination. If homogametic females can be easily identified there is potential to use them as parents to yield completely sexually uniform heterogametic female offspring [38]. Although some markers associated with sex determination have been identified [38], little is known about candidate genes, mechanism or map regions associated with the sex of crustaceans.

Here we undertake the first comprehensive genome scan for QTL associated with resistance to WSSV and for the sex-determining locus in *P. monodon*. A new WSSV challenge testing protocol that aims to deliver more accurate disease resistant phenotypes is devised and utilised. A set of 3959 linkage mapped transcribed gene SNPs are used to genotype 1038 sexed individuals derived from 7 full-sibling families challenged-tested for WSSV.

## Results

### Challenge tests

**Figure 1:**
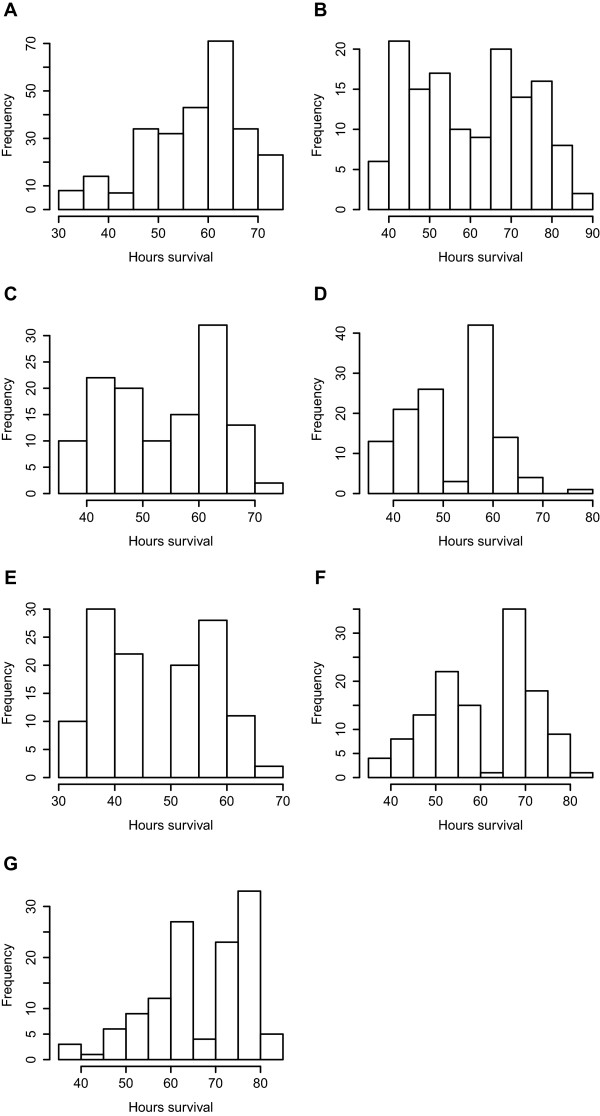
**Plot of hours of survival among progeny genotyped from 7 full-sibling families (A-G).**

Shrimp survived on average 57.2 ± 12.0 SD hrs post challenge and a spread of *hours of survival* was observed within families (eg. ranging between 30 and 90 hours for the upper and lower 40 percentiles genotyped from families B, F and G Figure 1). No mortality was observed in the control group injected with saline buffer.

### Genetic parameters associated with white spot syndrome virus resistance

Neither sex nor time of challenge (family) had significant effects on time to death in the model (Table 1, all 95% confidence intervals overlap zero).

**Table 1:** **Summary of MCMCglmm analysis under an animal model of days survival after WSSV experimental challenge**

		95% confidence limits			
Parameter	Mean	Lower	Upper	Effective sample	pMCMCglmm
(Intercept)	57.4	44.7	68.0	1522	<7e-04***
sexM	0.3	-1.0	1.7	1400	0.679
pedDS	3.1	-13.9	18.4	1400	0.64
pedES	-3.6	-17.9	14.9	1400	0.59
pedFS	-5.7	-20.8	11.6	1400	0.406
pedGS	-9.3	-24.6	8.4	1400	0.196
pedHS	9.5	-9.0	24.7	1400	0.201
pedHSa	3.4	-12.4	19.7	1585	0.6

Sex (male sexM) and family (pedDS, pedES, pedFS, pedGS, pedHS and pedHSa) fitted as fixed effects. Mean, mean of posterior distribution.

***P < 0.001.

### Linkage disequilibrium

Mean and median values of LD, measured as r^2^ between adjacent markers for the 3961 genome-wide distributed SNPs used in this study, were 0.35 and 0.30, respectively.

### QTL for WSSV resistance – GWAS and interval mapping analysis

The quality control steps excluded all markers with non-Mendelian inheritance and all individuals unassigned with parentage analysis, leaving 3959 markers and 1038 individuals for analysis. For the FASTA and GRAMMAS GWAS analysis the additional quality control steps excluded 135 markers and 17 individuals with a call rate of less than 95%, 5 individuals with high autosomal heterozygosity (FDR <1%) and 4 individuals with identity by descent ≥0.95. No markers or individuals with a call rate less than 0.1 and minor allele frequency <0.24% were detected. After the quality control, 3824 markers and 1019 individuals were selected for the FASTA and GRAMMAS analysis.

**Figure 2:**
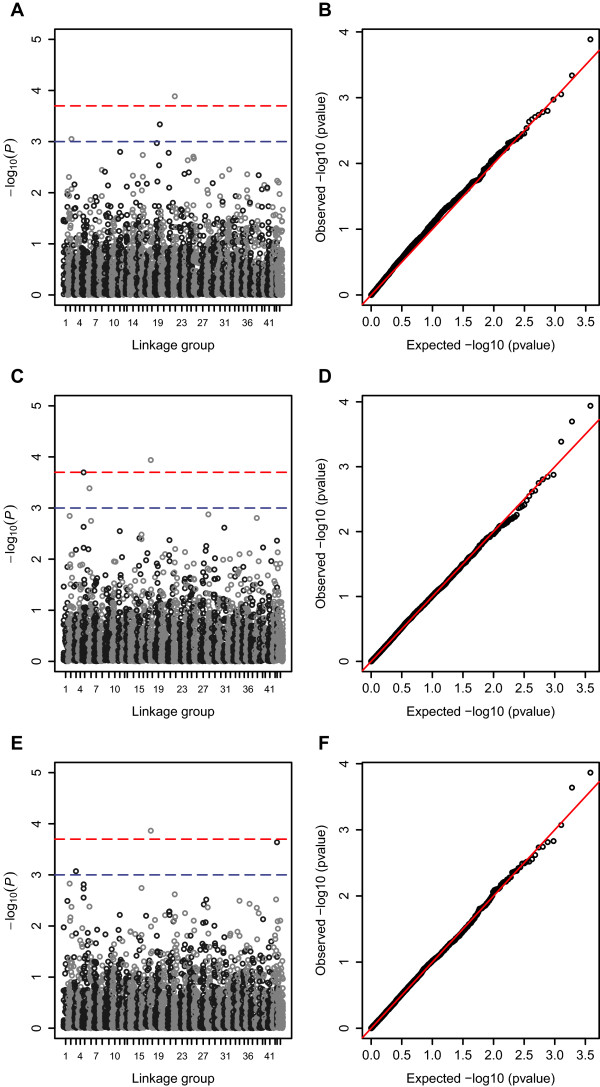
**Manhattan (A, C and E) and QQ plots (B, D and F) for GWAS analyses showing corrected** ***P*** **-values with 1 degrees of freedom after permutation testing for SNPs across the 44 linkage groups for trait** ***hours of survival*** **for tests QFAM (A and B), FASTA (C and D) and GRAMMAS (E and F).** Linkage positions are shown in centimorgons (cM) on the horizontal axis. Upper and lower dotted lines mark significance thresholds after Bonferroni correction of *P* < 0.01 and *P* < 0.05 respectively.

**Figure 3:**
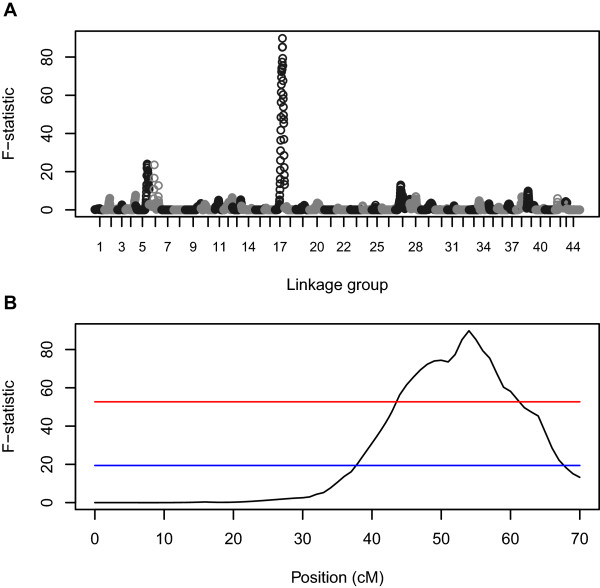
**GridQTL interval mapping F-test statistic plots for trait** ***hours of survival*** **across all linkage groups (A) and across LG17 (B).** Upper and lower dotted lines mark significance thresholds after permutation testing of *P* < 0.01 (genome-wide significance after Bonferroni correction) and *P* < 0.05 (chromosome-wide significance) respectively (plot B). Chromosome-wide significance was detected on linkage groups 5, 6 and 27 while genome-wide significance was detected on LG17.

**Figure 4:**
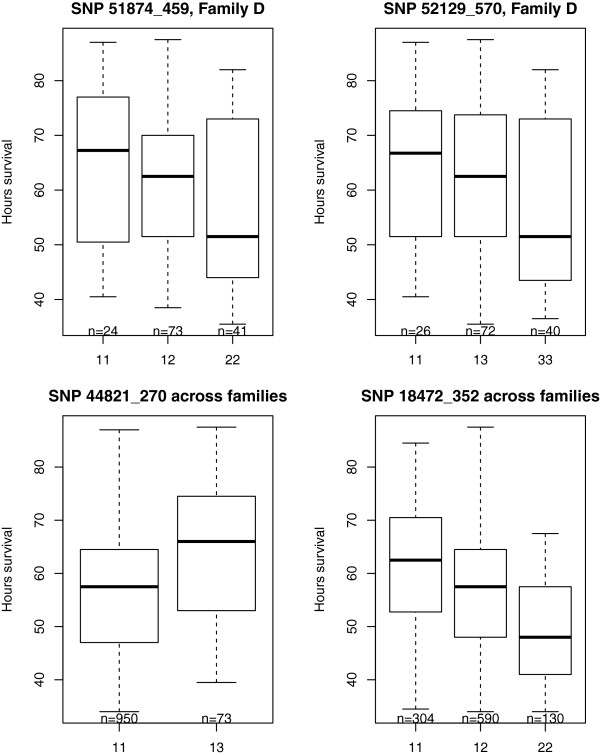
**Box plot showing** ***hours of survival*** **post-WSSV infection for genotypes detected at SNP loci 51874_459 and 52129_570 positioned at 51 and 50 cM respectively on linkage group 17 (mapping closely to the predicted QTL location at 54 cM) and across families at GWAS significant (** ***P*** **< 0.01) SNP loci 44821_270 on linkage group 18 and 18472_352 on linkage group 22.** Data is presented for family D in which both parents were heterozygous for the QTL on LG17.

Ten significant QTL for WSSV resistance (*hours of survival* post-WSSV infection, *P* < 0.05 after Bonferroni correction) were detected on linkage groups 2, 3, 5, 6, 17, 18, 19, 22, 27 and 43 (Table 2, Figures 2 and 3). Eight SNPs (51997_2402, 41442_21, 45605_1545, 29124_228, 44821_270, 50096_1789, 18472_352, 27976_64 on linkage groups 2, 3, 5, 6, 18, 19, 22, and 43 respectively) showed significant genome wide associations, and three regions (between SNPs 50756_3741 and 46539 on LG6, SNPs 25133_74 and 36717_243 on LG17 and SNPs 18687_338 and 3729_523 on LG27) showed significant linkage with *hours of survival*. These SNPs occurred in transcripts for genes encoding runt protein 1a, flagellar hook-length control protein, ubiquitin domain-containing protein ubfd1, paired-like homeodomain transcription factor 3, ankyrinn repeat and many unannotated genes. Box plots of *hours of survival* post-WSSV infection for individuals with alternative genotypes for two informative SNPs in the vicinity of the QTL detected on linkage group 17, and for GWAS significant SNPs on linkage groups 18 and 22 (Figure 4), show patterns indicating additive gene effects for these QTL.

**Table 2:** **Suggestive and significant QTL for trait** ***hours of survival*** **after WSSV challenge detected using PLINK (QFAM total) and GenAbel (FASTA and GRAMMAS) analyses in 7** ***P. monodon*** **families**

LG	Pos	SNP	Test	N	Effect	Stat	P-value	Sig	GeneID
1	60.3	39454_862	GRAMMAS	1007	1.14(0.53)	4.66	0.0032		unknown
2	0	47460_2015	QFAM	1024	-4.538	56.81	0.0094		dna p58 subunit
2	21.6	52022_4578	QFAM	1020	6.351	83.1	0.0049		plasminogen activator inhibitor 1 rna-binding protein
2	24.5	50149_330	FASTA	983	3.32(1.03)	10.49	0.0014		thyroid transcription factor 1-associated protein 26-like protein
2	24.5	50149_330	GRAMMAS	983	1.65(0.71)	5.44	0.0015		thyroid transcription factor 1-associated protein 26-like protein
2	30.5	52064_948	GRAMMAS	1007	0.95(0.49)	3.79	0.008		polymerase I polypeptide 194kda
2	36.6	36607_579	GRAMMAS	1006	-0.91(0.46)	3.94	0.0068		unknown
2	53.5	28698_101	GRAMMAS	965	-1.01(0.48)	4.4	0.0042		unknown
2	53.5	28698_101	FASTA	965	-1.75(0.65)	7.22	0.0082		unknown
2	61.8	51997_2402	QFAM	1023	-5.49	139.2	0.0009	*	runt protein 1a
2	62.5	35650_1855	FASTA	1004	2.11(0.79)	7.23	0.0081		unknown
2	62.5	35650_1855	QFAM	1018	5.408	100.5	0.0085		unknown
3	14.6	38676_1386	QFAM	1021	-4.2	41.79	0.0069		actin-binding rho-activating
3	29.5	41442_2163	GRAMMAS	1006	-1.35(0.55)	5.99	0.0008	*	flagellar hook-length control protein flik
3	29.5	41442_2163	FASTA	1006	-1.88(0.68)	7.62	0.0066		flagellar hook-length control protein flik
5	21.2	45605_1545	FASTA	1007	7.34(1.94)	14.26	0.0002	*	ubiquitin domain-containing protein ubfd1
5	21.2	45605_1545	GRAMMAS	1007	2.42(1.06)	5.22	0.0018		ubiquitin domain-containing protein ubfd1
5	21.9	35133_160	FASTA	991	-2.98(0.96)	9.56	0.0023		unknown
5	21.9	35133_160	GRAMMAS	991	-1.42(0.65)	4.81	0.0028		unknown
5	22.3	44076_3116	GRAMMAS	1004	-1.15(0.49)	5.4	0.0015		vacuolar proton atpase
5	22.3	44076_3116	FASTA	1004	-1.82(0.65)	7.79	0.006		vacuolar proton atpase
5	87	45237_316	GridQTL	1024	11.96(2.44)	24	0.0048	*	
5	96.5	30527_111	FASTA	992	-1.88(0.68)	7.67	0.0064		unknown
6	17.3	29124_228	FASTA	1007	4.82(1.34)	12.88	0.0004	*	paired-like homeodomain transcription factor 3
6	17.3	29124_228	GRAMMAS	1007	1.49(0.71)	4.42	0.0042		paired-like homeodomain transcription factor 3
6	39	50756_3741–46539_1081	GridQTL	1024	11.25(2.32)	23.54	0.0098	*	
6	42.8	33044_1018	FASTA	1007	3.65(1.15)	10.07	0.0018		erythrocyte band 7 integral membrane protein
8	45.4	52776_1335	QFAM	1021	5.849	56.79	0.0036		abb73282reverse transcriptase
9	10.4	42679_345	QFAM	1007	4.823	81.8	0.0039		unknown
9	59.9	48064_77	QFAM	1024	4.807	126.6	0.0072		unknown
11	24.7	60951_72	FASTA	1007	-3.46(1.14)	9.2	0.0028		unknown
11	24.7	60951_72	GRAMMAS	1007	-1.37(0.68)	4.05	0.0061		unknown
11	38.1	46551_1072	QFAM	1024	5.642	135.6	0.0016		multidrug resistance-associated protein 14
11	59.4	23272_344	FASTA	1007	3.52(1.33)	7.01	0.0091		26 s protease regulatory subunit
13	18	29098_2532	QFAM	1024	3.95	50.35	0.0076		actin-binding homolog 1
14	49.5	40042_2041	QFAM	1021	4.192	46.08	0.0046		unknown
15	27.2	32667_1134	QFAM	1023	-5.196	104.9	0.0067		usick-kaufman syndrome
15	47.8	44399_644	FASTA	1007	2.28(0.78)	8.61	0.0039		unknown
16	11	42291_720	GRAMMAS	1007	2.13(0.93)	5.24	0.0018		adp-ribosylation factor-like 2 binding protein
16	11	42291_720	FASTA	1007	4(1.34)	8.91	0.0033		adp-ribosylation factor-like 2 binding protein
16	11	38195_1528	FASTA	1003	2.46(0.84)	8.52	0.0041		fanconi anemia group a protein homolog
16	23.4	45647_100	QFAM	1018	-4.662	56.63	0.009		glutamyl-trna amidotransferase subunit
16	38.1	35920_135	QFAM	1012	3.142	43.9	0.0018		unknown
16	39.2	5999_123	QFAM	1024	6.677	92.39	0.0049		unknown
17	8.3	39727_708	GRAMMAS	1007	0.82(0.41)	4.01	0.0063		unknown
17	26.7	26178_2213	QFAM	1018	-4.67	78.32	0.0067		bobby sox
17	29	47941_2759	FASTA	1007	2.75(1.01)	7.34	0.0076		alsin isoform 2
17	54	25133_74 to 36717_243	GridQTL	1024	22.90 (2.42)	89.81	0.0001	**	
18	15.1	44821_270	FASTA	1006	7.26(1.85)	15.34	0.0001	**	unknown
18	15.1	44821_270	GRAMMAS	1006	3.35(1.2)	7.83	0.0001	**	unknown
18	81.5	24411_90	GRAMMAS	1006	-0.9(0.46)	3.83	0.0076		unknown
19	34.8	35006_276	QFAM	1024	5.931	92.83	0.0011		alanyl-trna synthetase
19	44.5	14555_138	QFAM	1021	6.616	125.5	0.006		unknown
19	70.9	51029_2543	QFAM	1023	-3.578	40.82	0.0029		insulin receptor substrate 1
19	82.4	50096_1789	QFAM	1021	5.243	77.12	0.0005	*	ankyrin repeat
20	23.1	36484_493	FASTA	1007	3.48(1.31)	7.01	0.0091		mitochondrial ribosomal protein l2
20	63.1	42447_399	GRAMMAS	1007	-1.14(0.57)	4.04	0.0062		unknown
20	63.1	42447_399	FASTA	1007	-2.26(0.84)	7.26	0.008		unknown
21	20.1	47262_891	GRAMMAS	1007	-0.93(0.45)	4.19	0.0053		myostatin 1b
21	20.1	47262_891	FASTA	1007	-1.75(0.66)	7.17	0.0084		myostatin 1b
21	20.1	47262_891	QFAM	1024	-4.787	82.96	0.0017		myostatin 1b
21	26	30265_1829	FASTA	1007	1.96(0.68)	8.44	0.0042		unknown
21	26	30265_1829	GRAMMAS	1007	0.88(0.43)	4.2	0.0052		unknown
21	28.5	29404_373	GRAMMAS	1007	1.26(0.63)	4.05	0.0061		unknown
21	28.8	19638_158	QFAM	1011	4.032	45.59	0.008		unknown
21	89.5	40988_772	GRAMMAS	1007	-0.98(0.5)	3.77	0.0082		c12orf66-like
22	9.1	52229_3858	GRAMMAS	1003	-1.08(0.55)	3.8	0.0079		nucleolar pre-ribosomal-associated protein 1-like
22	20.8	25410_46	GRAMMAS	986	-1.13(0.51)	4.96	0.0024		unknown
22	27.9	18472_352	FASTA	1007	-2.11(0.76)	7.69	0.0063		unknown
22	27.9	18472_352	GRAMMAS	1007	-0.98(0.49)	3.97	0.0066		unknown
22	27.9	18472_352	QFAM	1024	-5.815	104.9	0.0001	**	unknown
23	83.5	41044_732	QFAM	1024	5.885	110.7	0.0043		unknown
24	0.4	49156_279	GRAMMAS	1007	1.11(0.55)	4.13	0.0056		haspin
24	0.4	49156_279	FASTA	1007	2.21(0.81)	7.43	0.0073		haspin
24	50.3	51251_2007	QFAM	1018	-5.541	141.1	0.0023		cub-serine protease
25	0	44977_264	QFAM	1024	4.927	61.93	0.004		unknown
26	0.6	52048_2568	GRAMMAS	992	-1.12(0.57)	3.8	0.0079		adenosine monophosphate-protein transferase ficd homolog
26	0.6	52048_2568	QFAM	1009	-6.31	89.68	0.002		adenosine monophosphate-protein transferase ficd homolog
26	8.5	44451_587	QFAM	1023	5.597	76.98	0.0021		unknown
26	58.9	33059_367	QFAM	1024	-5.422	69.94	0.0059		unknown
27	40	18687_338-33729_523	GridQTL	1024	8.64(2.39)	13.04	0.018	*	
27	52.7	47625_1438	GRAMMAS	1006	-0.99(0.52)	3.7	0.0087		unknown
27	63.6	33004_1869	GRAMMAS	1007	-1.26(0.59)	4.51	0.0038		unknown
27	91.9	43302_1775	FASTA	1007	2.2(0.8)	7.54	0.0069		dead box atp-dependent rna helicase
27	91.9	43302_1775	GRAMMAS	1007	0.98(0.51)	3.73	0.0085		dead box atp-dependent rna helicase
27	101.7	49263_1068	GRAMMAS	1007	1.67(0.77)	4.73	0.003		unknown
27	101.7	49263_1068	FASTA	1007	3.12(1.11)	7.94	0.0055		unknown
28	20.8	51400_2931	GRAMMAS	1006	1.32(0.63)	4.38	0.0043		unknown
28	20.8	51400_2931	QFAM	1019	4.917	43.37	0.0089		unknown
28	30.6	47112_509	FASTA	1006	-2.82(0.87)	10.63	0.0013		chorion peroxidase
29	29.7	52042_128	QFAM	1022	4.654	94.7	0.0065		multiple c2 domain and transmembrane region
29	44	43412_2186	GRAMMAS	1007	-1.35(0.71)	3.58	0.0099		gpi-anchor transamidase
29	53.7	32409_114	FASTA	1005	1.86(0.71)	6.92	0.0096		unknown
30	77.3	51299_1729	QFAM	1016	4.115	60.01	0.0047		breast carcinoma-amplified sequence 3 homolog isoform 1
31	14.7	36096_367	FASTA	1007	3.82(1.24)	9.49	0.0024		nucleostemin-like protein
32	36.6	47777_1061	FASTA	1002	-1.82(0.7)	6.85	0.01		exonuclease 3–5 domain-containing protein 2 isoform 1
32	36.6	47777_1061	QFAM	1012	-4.716	76.83	0.0035		exonuclease 3–5 domain-containing protein 2 isoform 1
34	32.3	24101_537	GRAMMAS	1007	-1.56(0.72)	4.67	0.0032		zinc finger protein 64-like
34	32.3	24101_537	FASTA	1007	-2.68(0.99)	7.25	0.008		zinc finger protein 64-like
36	29.6	30057_491	QFAM	1023	-5.252	75.55	0.009		unknown
36	32.1	49829_3826	QFAM	962	-4.221	71.99	0.0044		unknown
36	57.6	50839_3313	GRAMMAS	1007	1.72(0.81)	4.54	0.0037		transcriptional enhancer factor tef-
36	57.6	50839_3313	FASTA	1007	3.08(1.14)	7.33	0.0077		transcriptional enhancer factor tef-
38	36.1	35013_386	FASTA	1007	-2.31(0.72)	10.32	0.0016		unknown
38	36.1	35013_386	GRAMMAS	1007	-0.88(0.42)	4.36	0.0044		unknown
38	66.9	17589_451	GRAMMAS	1004	1.67(0.82)	4.12	0.0056		unknown
39	0.2	35101_271	QFAM	1021	4.295	57.77	0.0045		unknown
39	51.2	49386_1117	QFAM	1024	-5.859	109.8	0.0086		phospholipase c gamma
39	59.4	36972_442	FASTA	1004	-1.85(0.66)	7.82	0.0059		unknown
39	59.4	36972_442	GRAMMAS	1004	-0.83(0.42)	3.86	0.0074		unknown
40	22.9	51885_4402	QFAM	1024	5.687	50.84	0.007		chromodomain-helicase-dna-binding protein 1
40	68.1	11637_107	QFAM	1020	-7.046	129.7	0.0083		non-lysosomal glucosylceramidase
41	1.6	26900_757	QFAM	1021	-7.651	113.7	0.0079		unknown
42	59	35645_15	GRAMMAS	1005	0.99(0.46)	4.73	0.003		unknown
43	0.4	27976_64	GRAMMAS	990	-1.4(0.52)	7.3	0.0002	*	unknown
43	0.4	27976_64	FASTA	990	-1.84(0.64)	8.39	0.0044		unknown
44	0	38601_555	FASTA	1007	-2.39(0.88)	7.41	0.0074		unknown
44	0	38601_555	GRAMMAS	1007	-1.1(0.57)	3.77	0.0081		unknown
44	3.2	42369_480	QFAM	1024	-5.011	91.99	0.0058		tbc1 domain family member 14 isoform a
44	26	51212_1738	QFAM	1024	6.066	107	0.0063		sodium bicarbonate transporter-like protein 11
44	40.4	20208_30	GRAMMAS	1007	1.55(0.79)	3.8	0.0078		unknown

LG, linkage group; Pos, location on LG in centimorgans; N, number of progeny and parents analysed; Effect, allele substitution effect of the minor allele with standard error in parenthesis (FASTA, GRAMMAS and GridQTL), Beta (QFAM); Stat, test statistic linear regression coefficient for QFAM, chi-square with one degree of freedom for FASTA and GRAMMAS analyses, F-statistic for GridQTL; P, point-wise empirical *P*-value (QFAM), permuted *P*-value with one degree of freedom corrected for inflation factor lambda (FASTA and GRAMMAS) or chromosome-wide *P* search with permutation and bootstrap analysis (GridQTL); Sig, significance after Bonferroni correction (*P < 0.05; **P < 0.01). GeneID, closest SNP homology from BLAST. Tests were considered suggestive when *P* < 0.01 before Bonferroni correction.

Some of the SNPs associated with QTL were found to map within or close to genes with putative immune functions of interest (Tables 2 and 3, Additional file 1). For example, the SNP marking a QTL at position 61.8 cM on linkage group 2 (51997_2402, *P* < 0.05 after Bonferroni correction for the QFAM test), occurred in a transcript that shared high homology to a gene encoding runt protein 1a in the signal crayfish *Pacifastacus leniusculus.* SNP 24034_664 at 47.3 cM on LG 2 in a transcript with homology to the proteasome (macropain) 26 s gene maps in the middle of a broad 41 cM region containing several SNPs in transcripts showing suggestive and significant associations with *hours of survival* after WSSV infection (including SNP 51997_2402, *P* < 0.05 after Bonferroni correction, at 61.8 cM). Variation at SNP 45605_1545 in a transcript with homology to a gene encoding ubiquitin domain-containing protein ubfd1 on LG 5 was associated with *hours of survival* (FASTA *P* < 0.05 after Bonferroni correction). The SNP 40050_2030 occurs in a transcript with homology to a gene encoding 26 s proteasome subunit s9 and maps 4.4 cM from SNP 33044_1018 (suggestive association on LG6) and 0.6 cM from a predicted QTL (GridQTL, position 39 cM, *P* < 0.05 chromosome-wide significance). The SNP 49912_5110 which occurs in a transcript with homology to the mitogen activated protein kinase gene, mapped 2.3 cM from the QTL position detected by GridQTL analysis on LG17 (*P* < 0.01 genome-wide significance). The SNP 52376_14757 occurs in a transcript that is homologous to the hect e3 ubiquitin gene and maps 2.4 and 3.9 cM from SNPs 51029_2543 (suggestive association) and 50096_1789 (significant association *P* < 0.05 after Bonferroni for the QFAM test) at 70.9 and 82.4 cM respectively along LG19. SNPs 48349_91, which occurs in a transcript with homology to proteasome (macropain) 26 s non- 2 gene, and 38683_977, which occurs in a transcript with homology to a gene encoding ubiquitin conjugating enzyme 7 interacting protein, map 12.8 and 0.6 cM respectively from SNP 50096_1789 (*P* < 0.05 after Bonferroni correction, test QFAM) at 82.4 cM on LG19. Three genes encoding proteins with putative immune function map near to SNP 18472_352 (*P* < 0.01 after Bonferroni correction, QFAM test) at 27.9 cM on LG22, SNP 52279_11861 which also maps to 27.9 cM on LG 22 and which occurs in a transcript with homology to the serine-threonine protein kinase gene, SNP 42578_2554 which occurs in a transcript with homology to a gene encoding mitogen-activated protein kinase-binding protein 1 which is 1.9 cM distant and SNP 50961_705 which occurs in a transcript showing homology to a gene encoding IGF2 mRNA binding protein and is 2.4 cM distant.

**Table 3:** **SNPs with homology to genes of putative immune function mapping near to QTL regions**

QTL		Closely mapping SNPs with putative immune function						
LG	cM	cM	SNP	GeneID	Length	Hits	E-value	Similarity
2	0, 21.6, 24.5, 30.5, 36.6, 53.5, 61.8* and 62.5	47.3	24034_664	proteasome (macropain) 26 s	991	20	3.48E-45	62.15%
		61.8	51997_2402	runt protein 1a	2649	2	5.91E-53	84.00%
5	21.2*, 21.9, 22.3, 87* and 96.5	21.2	45605_1545	ubiquitin domain-containing protein ubfd1	1764	20	5.32E-81	66.7%
6	17.3* 39* and 42.8	38.4	40050_2030	26 s proteasome subunit s9	2299	20	2.33E-143	76.50%
9	10.4 and 59.9	59.7	42539_708	E3 ubiquitin-protein ligase RAD18	1522	20	1.62E-51	46.90%
		59.9	37682_953	complement component	1318	20	1.69E-139	67.30%
11	24.7, 38.1 and 59.4	20.8	44253_2858	ubiquitin protein ligase	3938	20	0	64.25%
		38.4	42465_201	mitogen-activated protein kinase organiser 1	853	20	2.33E-57	58.65%
		59.4	23272_344	26 s protease regulatory subunit	1579	20	0	87.45%
15	27.2 and 47.8	27.7	17687_140	proteasome subunit alpha type-7	965	20	4.28E-101	90.05%
16	11, 23.4, 38.1 and 39.2	38.1	52008_2116	serine threonine-protein kinase 17b	3652	20	7.03E-39	83.60%
17	8.3, 26.7, 29 and 54**	5.6	50459_2444	interleukin enhancer-binding factor 2	2645	20	0	86.70%
		26.7	45405_1355	stress-induced-phosphoprotein 1 (Hop or HSP70-HSP90 organising protein)	3508	20	0	70.90%
		29.6	51513_1353	ubiquitin conjugation factor e4	4762	20	0	68.60%
		56.3	49912_5110	mitogen activated protein kinase	7539	20	7.36E-150	75.90%
19	34.8, 44.5, 70.9 and 82.4*	28	35516_4536	hect e3 ubiquitin	4790	20	0	66.25%
		37.8	47403_548	heat shock protein isoform a	1612	20	6.27E-25	64.90%
		68.5	52376_14757	hect e3 ubiquitin	16975	20	0	80.55%
		81.8	38683_977	ubiquitin conjugating enzyme 7 interacting protein	1048	20	8.96E-79	64.65%
		95.2	48349_91	proteasome (macropain) 26 s non- 2	3200	20	0	74.85%
21	20.1, 26, 28.5, 28.8 and 89.5	80.7	46753_1347	e3 ubiquitin-protein ligase shprh	1490	20	3.22E-105	70.25%
22	9.1, 20.8, 27.9**	26	42578_2554	Mitogen-activated protein kinase-binding protein 1	2589	20	0	79.6%
		27.9	52279_11861	Serine threonine-protein kinase smg1	14868	20	0	54.7%
		30.3	50961_705	IGF2 mRNA binding protein	6075	20	3.02E-138	68.90%
24	0.4 and 50.3	44.9	51084_1046	ubiquitin-conjugating enzyme e2 c	3227	20	2.73E-63	80.10%
25	0	4.7	51361_1388	inhibitor of kappa light polypeptide gene enhancer in b-kinase complex-associated protein	4354	20	1.74E-116	57.05%
28	20.8 and 30.6	12.6	30698_651	map kinase-activated protein kinase 2-like isoform 2	1544	20	6.12E-139	84.45%
29	29.7, 44 and 53.7	44	49666_3836	26 s proteasome non-atpase regulatory subunit 11-like	4562	20	3.62E-64	60.15%
32	36.6	37.5	49114_4840	ubiquitin carboxyl-terminal hydrolase 47	6432	20	0	65.70%
43	0.4*	2.2	45153_220	aax63905c-type lectin protein	1167	20	8.30E-15	43.75%

*P <0.05; **P <0.01 after Bonferroni correction. GeneID, identity allocated by blast2go using consensus annotations for the top hits. Length, length of query contig sequence. Hits, number of sequences found to match query (maximum 20). E-value, minimum e-value (probability of alignment occurring by chance) recorded for a hit. Similarity, percent mean similarity recorded across hits.

### Association with sex on LG30

**Figure 5:**
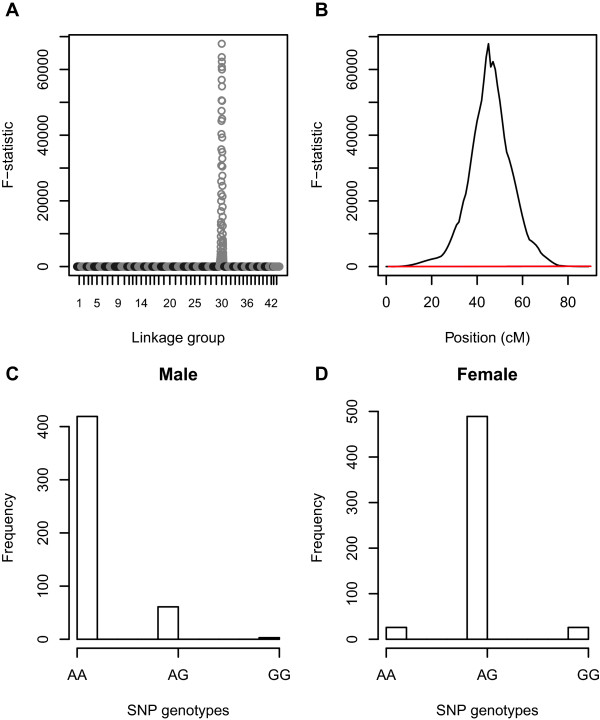
**GridQTL interval mapping F-statistic plots over all linkage groups (A) and on LG30 (B) for the trait** ***sex*** **, and genotype frequency differences between male (C) and female (D)** ***P. monodon*** **for SNP 49245_2916 located at 43.5 cM on LG 30 which was found to be significantly associated with sex (** ***P <*** **0.001 after Bonferroni correction).**

In all, 15 SNP markers were significantly associated with sex, (5 at *P* < 0.01 and 10 at *P* < 0.001 significance levels after Bonferroni correction, Additional file 2, Figure 5A and B). All significant associations mapped to a broad 43 cM interval of LG30 between positions 21.7 and 64.7 cM. The three markers with the strongest association mapped to an interval of 0.8 cM (positions 43.5 - 44.3 cM, SNPs 49245_2916, 49087_997 and 49482_526). Most significant was SNP 49245_2916 (*P* = 1.9E-49) which occurs in a gene encoding G7-c-like protein and von Williebrand factor A domain-containing protein 7 (Additional file 2). The sex locus was predicted to map to 45 cM on LG30 by the GridQTL interval mapping analysis (*P* < 0.001 genome-wide significance, Figure 5B).

The pattern of segregation of this locus to male and female offspring fits what would be expected for a locus associated with sex determination, assuming that female *P. monodon* are the heterogametic sex (Figure 5C and D). Eighty-seven percent of males (out of 483 genotyped) were homozygous AA for SNP 49245_2916 across the families (the allele frequency of A and G alleles was 0.93 and 0.07 respectively, n = 966) whereas ninety percent of females (541 genotyped) were heterozygous AG. Of the males that were not AA, 13% were AG, and less than 1% were GG genotypes. The GG males were only detected in one family (3/74 individuals in family 4 which also contained a high proportion, 30/74, of AG males). Most other families contained a low proportion of AG males, except two families (2 and 6) where all males were AA genotypes. Of the females that were not AG, 5% were AA and 5% were GG. The GG females were only detected in one particular full-sibling family (family 4 with 26/64 female genotypes recorded as GG). All families contained low numbers of AA females, except family 5 in which 56/56 females were AG. Also mapping to this region (at 44.3 cM) is SNP 43522_2279 which occurs in a transcript with homology to the feminisation-1 gene (fem-1 homolog c) in the nematode *Caenorhabditis elegans* (69% homology, contig length 3820 bases, Additional file 1 and Additional file 2).

## Discussion

Invertebrates rely on innate immune systems to recognise and respond to foreign agents. Resistance to disease is a complex quantitative trait that is likely to be regulated by the additive effects of many genes, epigenetics and by the environment. In contrast, sex, which is measured as a binomial qualitative trait, is likely to be determined by the action of a few genes mapping to a specific area of one linkage group. Variation affecting disease resistance or sex could act by changing the regulation of gene expression or by leading to modifications of the protein product and consequent function. The SNPs developed for this study were detected among shrimp sourced from the east coast of India and Andaman Islands [39]. In developing SNPs we included RNA from three individuals that had survived a severe WSSV outbreak on a farm in Bapatla. These surviving shrimp represented only 0.2% of the total post-larvae that were stocked for culture. They were later transferred to secure tank facilities where they lived for more than four months. These shrimp were found to be positive for WSSV using a nested PCR test. These survivors were included in the present study to improve the chance of detecting SNP variants that are associated with resistance to WSSV. All the SNPs used in the study occur in transcribed genes (ie. cSNPs).

The challenge test experiment used in this study which lodged shrimp in individual baskets was designed so that all shrimp could be collected and sampled within 1 hour of death and to prevent secondary infection (transmitted with cannibalism). Although the time from infection with WSSV to death is rapid, a controlled route of infection and dosage was chosen to prolong the overall time frame of the experiment as much as possible and to give a spread of *hours of survival*. Large full-sibling families (146 offspring per family on average) and frequent observation were also employed to give a strong power for detecting QTL.

Both the linkage and GWAS analyses detected significant QTL associated with *hours of survival* after WSSV infection. For three of the four QTL detected by linkage analysis, closely mapping SNPs with suggestive associations were detected by GWAS analysis (on linkage groups 5, 6 and 27, Table 2). Fewer QTL were detected using linkage analysis than using GWAS. While linkage analysis relies on the segregation of alleles within families, GWAS correlates the occurrence of SNP alleles with phenotypes across the population. Comparison of linkage analysis and GWAS has shown that GWAS, where all SNPs are fitted simultaneously as random effects, has greater power to discriminate linked QTL [40], especially those of limited or modest sized effects [41]. The sensitivity of linkage analysis is affected by the number of parents that are segregating for the QTL and neighbouring SNP loci and by the extent of linkage among SNPs mapping in the vicinity of the QTL. The sensitivity of GWAS depends on the existence of linkage disequilibrium between the QTL and single SNP loci (which, to some extent, is dependent on the number of SNPs tested) and on the existence of SNPs sharing a similar allele conformation to that of the QTL. It has been found by other studies that the two types of analyses generally yield inconsistent results, but can agree if the differences between the two methods (caused by differences in the precision for mapping QTL location, ability to account for multiple linked QTL and due to over estimation of what are sometimes modelled as fixed SNP effects), are accounted for [40].

For the GWAS analyses, the GRAMMAS and FASTA results were often in agreement, while the results of QFAM analysis were less often in agreement with GRAMMAS or FASTA. For instance, SNP 18472_352 on LG22 was found to be associated with *hours survival* by the QFAM test (*P* < 0.01 after Bonferroni correction), but was found to be suggestively associated with the trait by the GRAMMAS and FASTA tests. Similarly, SNP 51997_2402 on LG2 was associated with *hours survival* for the QFAM test (*P* < 0.05 after Bonferroni) and a closely mapping SNP was suggestively associated using the FASTA test. No agreement for the significant association detected by QFAM at position 82.4 cM was found by GRAMMAS or FASTA tests across LG19. Whereas, significant associations detected on linkage groups 3, 5, 18 and 43 by GRAMMAS or FASTA were supported by corresponding suggestive or significant associations by FASTA or GRAMMAS respectively for the same SNP. FASTA and GRAMMAS, which use genomic control to infer genetic relations from genomic data, and thereby account for the true genealogy (population structure and all levels of relationships), are thought to be superior to methods such as QFAM, which makes use of the observed genealogy (observed parent-offspring relationships in our study) [42].

### Candidate genes mapping to QTL regions

Several of the SNPs directly associated, or closely linked to WSSV resistance QTL, were found to occur in transcripts that share homology to genes with putative immune functions. Some of the genes, such as heat shock protein 21, c-type lectin and serine-threonine specific protein kinase, have been implicated in affecting the WSSV resistance of crustaceans in other studies [18, 20, 43–45]. Some are components of gene pathways, such as the ubiquitination pathway, which have been found to affect the pathogenesis of WSSV [46, 47].

### The ubiquitin proteasome pathway

The ubiquitin proteasome pathway has been shown to play an important role in immune defence and more specifically proteasome I is presumed to be involved in intracellular antibody-mediated proteolysis of antibody-bound viruses [48]. Six SNPs in transcripts with homology to proteasome encoding genes of interest were either directly or closely mapped to QTL for WSSV resistance (Table 3), including SNP 24034_664 in a transcript with homology to the proteasome (macropain) gene which was 14.1 cM from SNP 51997_2402 (*P* < 0.05 after Bonferroni correction, LG2), SNP 23272_344 in a transcript with homology to the 26 s protease regulatory subunit gene (suggestive association), SNP 40050_2030 in a transcript with homology to the 26 s proteasome subunit s9 gene which maps 0.6 cM from a QTL position predicted by linkage analysis (*P* < 0.05 chromosome-wide significance on LG6), SNP 17687_140 in a transcript with homology to the proteasome subunit alpha type-7 gene which was 0.5 cM from SNP 32667_1134 (suggestive association with *hours of survival* on LG15), SNP 48349_91 in a transcript with homology to the proteasome (macropain) 26 s non-2 gene which maps 12.7 cM from SNP 50096_1789 (*P* < 0.05 after Bonferroni correction, LG19) and SNP 49666_3836 in a transcript with homology to the 26 s proteasome non-atpase regulatory subunit 11-like gene which maps to the same position as SNP 43412_2186 (suggestive association at 44 cM on LG29).

Modulation of the host ubiquitin proteasome pathways by viral proteins is thought to affect viral pathogenesis, and four proteins have been identified in the WSSV (WSSV199, WSSV222, WSSV249 and WSSV403) [49–52] which interact with the *P. monodon* ubiquitination pathway (eg. with conjugating enzyme (E2) in shrimp) and act as viral E3 ubiquitin protein ligases to inhibit apoptosis and affect viral pathogenesis [46, 47]. Injection of recombinant *Fenneropenaeus chinensis* ubiquitin-conjugating enzyme E2 has been shown to reduce the mortality of shrimp challenged with WSSV, inhibit replication of WSSV and bind to (and ubiquitinate) WSSV RING domain-containing proteins [53], and ubiquitin C expression is up-regulated when *F. chinensis* are challenged by WSSV [54]. It follows that variation in the structure or expression of E3 ubiquitin-protein ligase, ubiquitin conjugating enzyme (E2) or other enzymes involved in the ubiquitin proteasome pathway, could be important in affecting the resistance or susceptibility of *P. monodon* to WSSV. Variation in a SNP in a transcript with homology to the ubiquitin domain-containing protein ubfd1 gene (45605_1545) mapping to 21.2 cM along linkage group 5 was found to be associated with WSSV resistance in this study (*P* < 0.05 after Bonferroni correction for the FASTA test). The SNPs in nine other transcripts with homology to genes involved in the ubiquitin proteasome pathway (two forms of e3 ubiquitin-protein ligase, two forms of hect e3 ubiquitin, ubiquitin carboxyl-terminal hydrolase 47, ubiquitin-conjugating enzyme e2 c, ubiquitin-conjugating factor e4, ubiquitin-conjugating enzyme 7 interacting protein and ubiquitin protein ligase, Table 3) were all found to show suggestive associations or to map closely to other SNPs significantly or suggestively associated with *hours of survival* after WSSV challenge in this study.

### Lymphocyte function and heat shock proteins

Interleukin enhancer-binding factor 2 is a transcription factor required for expression of the interleukin 2 gene which regulates the activity of lymphocytes responsible for immunity [55]. A SNP in a transcript with homology to the gene coding for this factor was found to map 3 cM from a SNP (3927_708) with suggestive association to *hours of survival* on LG17 (Table 3).

Heat shock proteins act as intercellular signalling molecules for the regulation of the immune response of many organisms, particularly with regard to lymphocyte mediated responses [56]. The Hsp70-Hsp90 organizing protein (Hop, SNP 45405_1355 at 26.7 on LG17, Table 3) is a co-chaperone that reversibly links HSP70 and HSP90, moderating chaperone activity. The expression of HSP70 and HSP90 increases in hemocyte and lymphoid organs when crustaceans (*Marsupenaeus japonicus* and *Procambarus clarkii*) are challenged with WSSV [43, 44]. HSP21 is normally highly expressed in *P. monodon* tissues, but is down-regulated following infection with WSSV [20].

### The TOLL pathway

Nuclear factor kappa-light-chain-enhancer of activated B cells (NF-kB, SNP 51361_1388 at 4.7 cM on LG 25, Table 3) is a rapid acting primary transcription factor which regulates the innate and adaptive immune cellular response to viral and other forms of infection. When pattern recognition toll-like receptors in T- or B-cells are activated, NF-kB enters the nucleus and up-regulates genes involved in development, maturation and proliferation (eg. type I interferon response genes). Large precursor molecules of NF-kB (p105 and p100) are processed by the ubiquitin/proteasome pathway which involves the degradation of ankyrin repeat c-terminal regions.

“Inappropriate” activation of NFKB has been linked to AIDS, whereas inhibition has been linked to disorders in immune cell development. The stimulation of activator protein 1 activity by mitogen-activated protein kinases is thought to elicit stress responses and promote cell survival and death in response to viral infection [57].

### Mitogen activated protein kinases

Protein kinase signal transduction pathways, including mitogen-activated protein kinases, have been shown to have important roles in the regulation of cytokine gene expression [58–60], particularly interleukin-1, which is a potent inflammatory cytokine regulating host defence and immune responses [61]. Mitogen activated protein kinases (MAP kinases) are involved in directing cellular responses to a range of stimuli including viral infection. Extracellular signal-regulated kinase is a type of serine-threonine specific protein kinase that is activated by WSSV in the early stage of infection, and when silenced or inhibited, reduces WSSV proliferation, and delays viral early gene transcription, in *L. vannamei* [45]. The SNPs in transcripts with homology to mitogen activated protein kinase, mitogen-activated protein kinase organising factor 1, map kinase-activated protein kinase 2-like isoform, serine-threonine protein kinase, interleukin enhancer binding factor and mitogen-activated protein kinase-binding protein 1 were found to map near to SNPs showing suggestive and significant (LG17 GridQTL *P* < 0.01 genome-wide significance) associations with *days survival* on linkage groups 11, 16, 17, 22 and 28 (Table 3 and Figures 2 and 3).

The mRNA binding proteins, such as IGF2 mRNA binding protein (gene mapping 2.4 cM from SNP 18472_352, *P* < 0.01 after Bonferroni for the QFAM test, Table 3), play an important role in stabilizing mRNAs during cellular stress [62].

### Lectin

Lectins are non-self-recognition factors thought to be involved in immune recognition and microorganism phagocytosis through opsonisation in crustaceans [63]. A SNP in a transcript with homology to C-type lectin (45153_220) maps 1.8 cM from SNP 27976_64 on LG43 (*P* < 0.05 after Bonferroni correction for the GRAMMAS test, Table 3). Tiger shrimp surviving more than 84 hrs post WSSV infection have been observed to have higher haemocyte expression of c-type lectin [18]. WSSV infected *L. vannamei* that are pre-challenged with WSSV shower higher haemocyte expression of c-type lectin than previously naïve individuals [17]. Lectin is also more highly expressed in the hepatopancreas of resistant *L. vannamei* [64], and in the haemocytes and hepatopancreas of resistant *M. japonicus* [65, 66], than more susceptible individuals. C-type lectin-like domains have been detected in other genes such as PmAV, which are believed to be involved in conferring viral resistance in *P. monodon* [33].

### Runt protein

The runt protein is up-regulated prior to haemocyte release and is known to be involved in haematopoiesis [67]. The RUNT-related transcription factors (eg. RUNX3/p33) play important roles in the development and differentiation of the immune system [68] and mutations in this gene are known to be associated with greater susceptibility to autoimmune disorders [69]. The expression of RUNT domain protein is 40% lower in Norwegian lobsters (*Nephrops norvegicus*) that are immunologically suppressed by high levels of manganese [70]. A SNP associated with WSSV resistance on LG2 (51997_2402, *P* < 0.05 after Bonferroni correction for the QFAM test, Table 2), occurred in a transcript which shared high homology to runt protein 1a in the signal crayfish *Pacifastacus leniusculus.*

### Detection of markers associated with sex

Although sex determination is a simply inherited binary trait in most organisms, the precise genetic processes affecting sex determination have been found to be complex and diverse. SNP 43522_2279 occurs in a transcript for a gene that shares homology with Feminization-1 (Fem-1), a known signal transducing regulator affecting sex determination in the nematode *Caenorhabditis elegans* [71, 72]. This gene maps to the same position (at 44.3 cM on LG30) as SNP 49482_526, is 0.7 cM from the position of the sex determining locus predicted by GridQTL and is sandwiched 0.5 cM from SNPs 48571_1638 and 46782_1391, and 0.8 cM from SNPs 49245_2916 and 49087_997, all of which are SNPS found to be significantly associated with sex (*P* < 0.001 after Bonferroni correction, Additional file 2, Figure 5B). FEM-1, FEM-2 and FEM-3 form a CUL-2-based ubiquitin ligase complex which promotes proteolysis of the male-repressing transcription factor TRA-1, which is a regulator of sex determination by ubiquitin-mediated proteolysis [73, 74]. FEM-1 is the substrate recognition subunit in the complex, while FEM-2 and FEM-3 act as cofactors [74]. Maternal FEM-1 transcripts have been shown to prevent epigenetic silencing of FEM-1, which is believed to help protect the identity and integrity of the germ line [75]. Comparative mapping was unable to verify whether this is the same region containing the AFLP marker developed by [38] for sexing *P. monodon*.

For SNP marker 49245_2916, which showed the strongest association with sex, most males were AA genotypes while most females were AG genotypes. However, for family 4 there was a high proportion of AG male offspring (30/74) and high proportion of GG female offspring (26/74). In this instance the male parent had marker genotype AG (but sex locus genotype ZZ) such that ZZ males were either genotypes AA or AG, and WZ females were either genotypes AG or GG, at the marker locus. Possible explanations for other discrepancies (eg. the low frequency occurrence of AA and GG females in other families) are that recombination between the marker and sex determining locus occurred, that more than one gene in this linkage group effects sex determination, that environmental conditions during development are also influencing sex determination and/or that some homogametic females naturally occur. These discrepancies highlight that use of a single SNP marker locus for identifying sex will not be possible until the causative mutations for sex determination are identified.

In summary, indications are that the markers identified by this study, could be useful for the purpose of identifying homogametic females. Detailed studies of mutations and phenotypes in candidate genes mapping in this region of linkage group 30, could lead us to a better understanding of the genetic mechanisms affecting sexual dimorphism in *P. monodon*. In other invertebrates such as *C. elegans* there are a diversity of molecules and control networks involved in sex determination [71]. The models for sex determination developed for *C. elegans* and other invertebrates such as *Drosophila melanogaster* will be informative.

### Application to marker assisted or genomic selection

Further research is needed to predict the most effective means of using the markers identified here to assist the genetic improvement of WSSV resistance. Consideration needs to be given to the overall goals of the breeding programs to which marker information is applied. In 2001, Meuwissen *et al.* [76] devised a method for the prediction of total genetic value using genome-wide dense marker maps, without phenotypic information, which has otherwise become known as genomic selection (GS). With the development of new low-cost fully-automated genotyping technologies, use of genome wide dense marker information is becoming more feasible for many species, especially for traits where direct measurement of the performance of individuals is problematic, such as disease resistance. GS uses information about genome-wide marker associations to estimate the breeding value of candidate individuals.

Validation using a population of tested and genotyped training individuals is necessary to estimate the effects at each genomic interval for GS. Effects estimated at numerous evenly spaced loci across the genome, including the QTL marker loci identified in this study, could be used to calculate genomic estimated breeding values for genomic selection. The weighting placed on each marker in the overall breeding value would depend on the relative allele substitution effect, and standard error, for each QTL (as shown in the *effect* column of Table 2) and on the emphasis placed on marker and/or phenotypic information for other traits included in the selection index. Estimation of these allele substitution effects differs, depending on the method and training populations used for their calculation. Linkage analysis within families tended to estimate higher allele substitution effects than GWAS across families (Table 2). Over-estimation of the size of the QTL effect was expected [77], particularly as selective genotyping was used in this study. Selective genotyping using sparse markers has been predicted to be effective for GS [78]. Higher emphasis for GS might be given to individuals inheriting favourable alleles at SNP marker loci such as 25133_74 where the estimate of the allele substitution effect is relatively large.

## Conclusions

From evidence in the available literature, genes affecting the action of the ubiquitin-proteasome pathway, lymphocyte-cell function, heat shock protein function, the TOLL pathway, protein kinase signal transduction pathways, mRNA-binding proteins, lectins and the development and differentiation of the immune system (eg. RUNT protein 1A), which were found in this study to closely map to SNPs on linkage groups 1, 2, 5, 6, 9, 11, 15, 17, 19, 21, 22, 24, 25, 28, 29, 32, and 43 suggestively or significantly associated with QTL affecting WSSV resistance in *P. monodon*, are all candidate genes that could be involved in controlling the immune response to this viral disease in this species. Sex is associated with the segregation of a number of SNPs mapping to linkage group 30. The strongest association with sex occurred for 3 SNPs mapping to a 0.8 cM stretch between positions 43.5 and 44.3 cM where the feminisation gene (FEM-1 in *C. elegans*) was positioned (44.3 cM). Interval mapping predicted that the QTL was positioned at 45 cM. The feminisation gene is known to be an important component of the CUL-2-based ubiquitin ligase complex and this complex is known to be involved in the control of sex determination in nematodes by promoting proteolysis of the male-repressing transcription factor TRA-1. Future efforts to identify the causative genes affecting these traits should focus on the fine mapping of genes in these regions and mutation experiments to elucidate function. This has been an effective strategy for livestock such as dairy cattle where genes affecting musculature [79] and milk composition [80, 81] have been identified. In the meantime, markers found to be associated with WSSV resistance could be applied to supplement genetic evaluations made by selective breeding programs for *P. monodon* (eg. run by Moana in Hawaii) and the efficacy of marker assisted selection for improving resistance to WSSV should be further evaluated in this and closely related species such as *L. vannamei*.

## Methods

### Shrimp sourced for challenge test experiments

Adult males and non-gravid female tiger shrimp from the wild were procured from the East coast of India and kept in the quarantine facility of the Muttukadu Experimental Station (MES) of Central Institute of Brackishwater Aquaculture, 35 km south of Chennai. These shrimp were checked for the presence of WSSV using a simple method to isolate the virus [82] and a WSSV detection kit (Bangalore Genei). The adults that were clear of WSSV, were eye-ring tagged and shifted to the maturation facility of the Crustacean Culture Division of MES for breeding trials. Two females and a male were placed together for mating in one tonne fibre re-inforced plastic (FRP) tanks. The shrimp were fed on a diet consisting of squid and polychaete worms which facilitates maturation. From maturation trials, seven full-sib families were produced. The shrimp from these families were cultured in separate hapas in a pond to an injectable size of about 3 to 5 g in order to retain family identity. At this stage, approximately 200 juveniles were randomly collected from the hapas and transferred to the challenge test facility where they were introduced into a 4 t concrete cement tank. The shrimp were allowed to de-stress for a couple of days to overcome the transportation stress. From each lot of 200 shrimps, a sample of ten shrimp were collected at random and tested using the WSSV detection kit.

### WSSV challenge experiment

A custom-made experimental facility, for preventing cannibalism, was fabricated for challenge studies to achieve recovery of all challenged shrimp. This facility consisted of multiple plastic baskets that were anchored to a support and lodged side-by-side at the same depth (just below the water surface) in a cement tank. Only one shrimp was housed in each basket during the experiment. Each basket had a lid for ease of placing or removing shrimp. The base of each basket had plastic wire mesh stitched to the sides such that feed pellets could be retained and faecal matter could easily pass through.

The muscle tissue from juvenile shrimp that were fed with WSSV-infected shrimp meat were used for extraction of WSSV virus following the protocol of [82]. The virus stock concentration was established as 1.04 X 10^6^ copies per μl in a real-time standard curve experiment. Trials were undertaken to compare intramuscular and oral routes of challenge and it was observed that intramuscular injection gave consistent results compared to the venocatch method. Consequent to this finding, all the experimental shrimp were challenged with the WSSV virus following the intramuscular method. The shrimp were injected intramuscularly with 100 μl of 10^-5^ dilution of virus stock using 1 mL tuberculin syringe. The virus was injected into the muscle tissue between the third and fourth abdominal segments on the lateral side. Extra care was exercised to avoid physical injury to the intestine and aorta running along the dorsal side and nerve cord running along the ventral side of the abdomen. After injection, the shrimp were retained in a 4 tonne cement tank for 6 hours to de-stress and to observe any mortality due to physical injury. De-stressed shrimp were then placed in individual baskets and monitored at hourly intervals for mortality. Simultaneously, twenty juvenile shrimp were injected with 100 μl of TNE (Tris–HCl-NaCl-EDTA) buffer solution and kept in a 100 L FRP tank. Care was taken to inject these shrimp first before challenging the test animals to avoid contamination. These shrimp served as a control and were kept under constant observation until the actual challenge experiment was completed. Each family was challenged on separate occasions. Care was taken to maintain uniform conditions for all individuals and families that were challenged. The salinity of the water, the weight of shrimp, the viral dose and the distribution of shrimp in baskets were similar for all the families.

Continuous aeration was provided for the experimental and control tanks. The animals were checked for mortality on an hourly basis. Water temperature was recorded on an hourly basis and pH and salinity was recorded once every morning. The water in the experimental and control tanks were exchanged daily (at 50%) when faecal matter and unused feed at the bottom of the tank was siphoned out in the process. Fresh seawater was provided after removing the debris at the bottom. The cleaning process was carried out daily until the last shrimp died.

When the challenged shrimp started dying, survival data (time to death) along with sex and wet weight of each shrimp were recorded. The dead shrimp were removed and stored at -80°C for DNA extraction.

### SNP markers and genotyping

Parents, along with the most susceptible and resistant 40 percentiles of progeny (based on *hours of survival* post-WSSV infection), were selected from each family for genotyping to find QTL. In all, 1024 offspring belonging to 7 full-sibling families that were challenge tested as described above, were successfully genotyped. Genomic DNA was extracted from the challenged shrimps using the Phenol Chloroform method as described by [83] with slight modifications. The quality of extracted DNA was checked on 2% agarose gel in 1X TBE buffer after electrophoresing at 50 V for an hour. The purity of DNA was checked using OD values at 260 and 280 nm. Quantification was achieved using OD value at 260 nm in Nanodrop 2000C (Thermo Scientific). The DNA of the experimental shrimp was extracted, dissolved in TE (Tris-EDTA) buffer, stored carefully in eppendorf tubes and transported in dry ice to Nofima, Norway for genotyping.

Genotyping was performed with 6 K custom developed Illumina Infinium iSelect Beadchips containing 6 K SNPs from *P. monodon* transcribed genes [39]. The SNPs were identified by two numbers separated by an underscore, where the first number identified the contig containing the SNP, and the second number was the SNP position in base numbers along the contig length. The same set of SNP genotypes and families used to detect QTL in this paper were previously used to construct a linkage map for *P. monodon* [39]. The sex averaged map consisted of 3961 informative SNPs which were assigned to 44 linkage groups. We used the map distances for the SNPs on the sex averaged map for the QTL analysis described below. The parentage of the challenge tested animals was checked when the linkage map was created [39].

### Genetic parameters, significance of fixed effects and correlation of traits

An animal model was applied to estimate genetic parameters (without accounting for SNP genotype). The animal model decomposed the phenotypic variance into additive genetic and environmental components. Our main interest was whether sex and/or time of challenge (family) should be included as fixed effects in the QTL analysis and whether weight should be included as a covariate. A Markov chain Monte Carlo (MCMC) method using a multi-trait generalised linear mixed effect model (glmm) in a Bayesian estimation framework, with animal breeding value and ID fitted as a random effects, was used for the analysis (R Package, MCMCglmm, [84], http://www.cran.r-project.org). The ID was the same as the animal factor, but was used by MCMCglmm to dissociate individual records from the pedigree and give an indication of between individual variance [85]. The model fitted was,


where *y* was time to death, sex and family were fitted as fixed effects, animal and ID were random animal effects and *mu* represented unknown random residual effects. A bivariate model (similar to the above) was used to obtain covariance components, and the genetic correlation between weight and time to death was estimated as,


where *σ*_*A*1*A*2_ is the estimated additive genetic covariance component between the two traits.

The model was run using 300,000 iterations as burn-in, 1 million iterations for sampling and a thinning interval of 500. A “plausible” prior assuming weak genetic control (additive genetic variance, permanent environmental variance and residual variance accounting for 0.2, 0.1 and 0.7) was used with the smallest possible degree of belief parameter (n = 1).

### Linkage disequilibrium

Linkage disequilibrium measured by r^2^ was calculated for all adjacent SNP pairs with the PLINK software package (Purcell et al., 2007).

### QTL for WSSV resistance – linkage analysis

Data were analysed using a regression-interval mapping method available through the web-based software GridQTL [86]. The sib-pair model was utilised in order to take advantage of the full-sib nature of the animal pedigree. Sex was included as a fixed effect, and weight included as a covariate in the model. *P*-values were calculated for all trait-by-LG combinations with the significance of the peak F-statistic (putative QTL) estimated after 10,000 chromosome-wide permutation tests. A QTL was found to be genome-wide significant if the chromosome-wide significance level was smaller than 0.0011 (0.05/44), a Bonferroni correction based on the number of linkage groups in *P. monodon*. This correction was equivalent to a Benjamini Hochberg [87] false discovery rate of >95% (*q*-value of 0.98), such that it was expected that more than 95% of the significant results actually were false positives. QTL were denoted as “suggestive” when *P* < 0.01 (before Bonferroni correction).

### QTL for WSSV resistance - GWAS

QTL GWAS analyses were performed in several ways. First we determined which markers and individuals should be excluded from the GWAS analysis using the check.marker function in GenABEL (http://www.genabel.org). This function was used to exclude individuals or markers with call rate <95%, markers with minor allele frequency <0.24%, individuals with high autosomal heterozygosity (FDR <1%) and individuals with identity by state ≥0.95. Genomic kingship was computed between all pairs of individuals. We performed a pedigree based association analysis where the pedigree is a confounder (where the heritable trait is more similar between close relatives and therefore some degree of association is expected between any genetic marker and any heritable trait). The effect of the confounding pedigree is expected to inflate the resulting null distribution of the chi square test statistic by a certain constant, lambda. Lambda is a function of the traits heritability and pedigree structure (expressed as a kinship matrix). Two fast tests for genome wide association were applied, Family-based Score Test for Association (FASTA, [88]) and Genome-wide Rapid Analysis using Mixed Models And Score test (GRAMMAS, [42]) using the R package GenABEL. A mixed polygenic model of inheritance was assumed in order to study association in our genetically homogeneous families where *hours of survival* (y) was modelled as


where μ was the intercept, G describes the polygenetic effect (contribution from multiple independently segregating genes all having a small additive effect on the trait) and e describes the random residual effects. The joint distribution of residuals in the pedigree was modelled using a multivariate normal distribution with variance-covariance matrix proportional to the identity matrix. A genomic kingship matrix, generated by calculating the average identity-by-state between individuals in the pedigree (ibs in GenABEL), was used as the relationship matrix for FASTA and GRAMMAS. Both FASTA and GRAMMAS exploit maximum likelihood estimates of the intercept from the polygenic model. One thousand permutations were used to estimate genome wide significance for both the FASTA and GRAMMAS tests. The *P*-value for the 1 degrees of freedom test was corrected for the inflation factor. Genomic control was applied by dividing the observed test statistic (*P*-value for the 1 degrees of freedom test) by the genomic inflation factor λ (where λ is the regression coefficient of the observed *χ*^2^ test statistic onto the expected *χ*^2^ test statistic). Genomic control is believed by some authors to circumvent the need for Bonferroni correction for multiple testing [89].

The QFAM analysis module in PLINK (http://pngu.mgh.harvard.edu/purcell/plink/ [90]) was used to perform a linear regression of phenotype on genotype. In this case the module used an adaptive permutation procedure to correct for family structure. Association testing was performed across the total data. Data from a total of 1024 offspring and 14 parents (7 nuclear families) were used with a genotyping success rate of 99%. Minimum number of permutations per SNP was 5, maximum 1 million, alpha level threshold 0, confidence interval on empirical p-value 0.0001 and intercept and slope of the pruning interval 1 and 0.001 respectively. GWAS associations with significance at *P* < 0.001, *P* < 0.01 and *P* < 0.05 levels after Bonferroni correction based on the number of linkage groups (which was 44 for *P. monodon*) were noted for all tests. GWAS associations were denoted as “suggestive” when *P* < 0.01 (before Bonferroni correction). As explained for the linkage analysis, the Bonferroni correction was equivalent to a Benjamini Hochberg [87] false discovery rate of >95% (*q*-value of 0.98).

### Mapping the sex-determining locus

SNPs significantly associated with sex were detected using a simple *χ*^2^ test of observed and expected allele frequencies in male and female offspring across families under the null hypothesis that the segregation of alleles would be independent of sex. Associations were treated as significant when *P* < 0.01 after Bonferroni correction based on the number of linkage groups. Regression interval mapping using the sib-pair module was also carried out in GridQTL as described for the WSSV analysis using sex as a phenotype.

### Availability of supporting data

The supporting high density *P. monodon* linkage map and SNP characterisations can be found in [39]. Annotated transcriptome sequence data is available through the Transcriptome Shotgun Assembly Database of NCBI (accession numbers JR196815 – JR235449, http://www.ncbi.nlm.nih.gov/Genbank). Other supporting data (map position and annotation for linkage mapped transcripts, tests for association with sex) are included in the additional files section.

## Electronic supplementary material

Additional file 1: **Map position and annotation for 3961 transcripts linkage mapped by** **[** [39]**].** LG, linkage group. cM, position of SNP on linkage group in centimorgans . GeneID, closest homology to contig from BLAST. Length, length of contig in number of bases. NumHits, number of BLAST matches above threshold (Karlin-Altshul cut off E-score of 0.001, maximum number of 20). MinEValue, Karlin-Altshul E-score. (XLSX 283 KB)

Additional file 2: **Map position and tests for association with sex for transcribed SNPs on LG30.** LG, linkage group. cM, position of SNP on linkage group in centimorgans. GeneID, closest homology to contig from BLAST. df, degrees of freedom. **, *P* < 0.01 after Bonferroni correction. ***, *P* < 0.001 after Bonferroni correction. (XLSX 17 KB)
